# Risk Factors for Death in Patients with Severe Acute Pancreatitis in Guizhou Province, China

**DOI:** 10.1155/2024/8236616

**Published:** 2024-04-01

**Authors:** Jun Li, Jingyan Gao, Min Huang, Xiaoyun Fu, Bao Fu

**Affiliations:** ^1^Department of Critical Care Medicine, Affiliated Hospital of Zunyi Medical University, Zunyi City, Guizhou Province, China; ^2^Severe Acute Pancreatitis Diagnosis and Treatment Center of Guizhou Province, Zunyi City, Guizhou Province, China; ^3^Department of Critical Care Medicine, The People's Hospital of Liupanshui City, Liupanshui, Guizhou Province, China

## Abstract

**Aim:**

To compare the clinical characteristics of survival and nonsurvival patients with severe acute pancreatitis (SAP) and explore the risk of mortality in SAP patients.

**Methods:**

This was a single-center retrospective study performed in a severe acute pancreatitis diagnosis and treatment center. According to the outcome, SAP patients were divided into survival group and nonsurvival group. One-way ANOVA or independent *t*-test was used to compare the clinical characteristics of two groups of patients. Multivariate retrospective analysis was used to identify risk factors for mortality in SAP patients.

**Results:**

A total of 486 SAP patients were included in the study, and the 90-day mortality for SAP patients was 13.58%. The common etiologies of SAP are biliary tract diseases (69.75%) and hyperlipidemia (17.28%). The most common complications caused by SAP were organ failure (55.14%), ARDS (50.62%), AKI (30.45%), sepsis (27.16%), and abdominal fluid collection (27.57%). There were differences in age, complications, and medical intervention between the nonsurvival group and the survival group. The main causes of death were infection (46.97%), abdominal bleeding (28.79%), and organ failure (9.09%). The binary logistic regression analysis showed that there were significant differences in age, AKI, sepsis, abdominal hemorrhage, organ failure, laparotomy, creatinine, and APTT between the nonsurvival group and the survival group.

**Conclusion:**

Age, AKI, sepsis, abdominal hemorrhage, and organ failure are risk factors for mortality in SAP patients. SAP patients with high creatinine and prolonged APTT upon admission require doctors to be vigilant. The main cause of death in SAP patients is pancreatitis-related organ failure and secondary infection.

## 1. Introduction

Acute pancreatitis (AP) is one of the common acute abdominal diseases that requires hospitalization in clinical practice. Despite all efforts to make early diagnosis and timely treatment of AP, the mortality remains high, ranging from 2% to 9% [1–3]. The severity of AP varies from mild to severe and fatal. The mortality of severe acute pancreatitis (SAP) can reach up to 30% and needs to be managed in ICU [4, 5]. A previous study showed that the mortality at hospital discharge was different among various etiologies of SAP [6]. There was a significant correlation between early enteral nutrition (EN) and reduced in-hospital mortality in biliary pancreatitis, while age and mechanical ventilation requirements were associated with increased mortality [6]. A recent study [7] found that respiratory failure was the most common cause of death in patients with alcoholic pancreatitis. Another study has shown that AKI is independently associated with higher mortality rates in AP [8]. Therefore, there are differences in the risk factors for SAP mortality among various studies.

This study was conducted at a diagnosis and treatment center for severe acute pancreatitis in Western China, located in a plateau region with Castro landforms and underdeveloped economy. People in this area prefer high-fat and spicy diets, with a high morbidity of AP. The exploration of the clinical characteristics and risk factors for mortality of SAP patients in this region will help clinicians understand the clinical characteristics of SAP in Western China.

## 2. Patients and Methods

The study was conducted in the Department of Critical Care Medicine at the Affiliated Hospital of Zunyi Medical University. The department is the diagnosis and treatment center for severe acute pancreatitis in Guizhou Province. SAP patients admitted to the center from January 2018 to December 2022 were included in the study. Any data that may reveal the identity of the patient was concealed.

The general information and treatment data of the patients were extracted from the electronic medical record system. The diagnosis of AP and SAP refers to the Atlanta classification criteria revised in 2012 [9]. The diagnostic criteria for AP are as follows: (1) persistent abdominal pain; (2) serum amylase and/or lipase levels that are three times higher than the normal upper limit; and (3) typical characteristic manifestations of abdominal imaging. SAP is defined as the occurrence of sustained single or multiple organ failure greater than 48 hours on an AP basis, or a Marshall score greater than 2. According to the etiology, SAP patients were divided into 6 groups, including biliary tract disease, hyperlipidemia, alcohol, trauma, mixed factors, and idiopathic. The diagnosis of biliary pancreatitis is mainly based on abdominal ultrasound, CT scan, magnetic resonance imaging, or endoscopic retrograde cholangiopancreatography (ERCP) [10]. Alcoholic pancreatitis is defined as drinking more than 50 grams of alcohol per day for more than 5 years, or the patient drinking excessively shortly before the onset of the disease, with other possible causes excluded [10]. If gallstones and/or excessive alcohol consumption are excluded and serum triglycerides are above 1000 mg/dL, she/he will be considered as hyperlipidemic pancreatitis. Mixed causes are considered to be compatible with at least two causes simultaneously. Idiopathic acute pancreatitis refers to pancreatitis whose etiology has not been determined after comprehensive screening.

All patients underwent chest and abdominal computed tomography scans 48 hours after admission, and the laboratory examinations were completed within 24 hours of admission. This study did not limit the age of the patients. Patients with incomplete clinical data or patients transferred to another hospital for treatment were excluded. The patient's data collection, etiology, and disease severity assessment were performed by two senior critical care physicians.

Continuous variables were shown as medians with interquartile ranges (IQR) or mean (standard deviation (SD)). Categorical variables were presented as percentages and compared using the chi-square test or Fisher's exact test. For quantitative variables, we used the Student's *t*-test or the Mann–Whitney *U* test. Multivariate retrospective analysis was used to assess risk factors for mortality in SAP patients, and *P* < 0.05 was considered statistically significant. Statistical analyses were performed using SPSS software (version 18.0, IBM Corp, Armonk, NY, USA.) and GraphPad Prism 5 (Dotmatics, Bishop's Stortford, UK).

## 3. Results

### 3.1. Comparison of Clinical Characteristics between Patients in the Survival Group and the Nonsurvival Group

**Figure 1:**
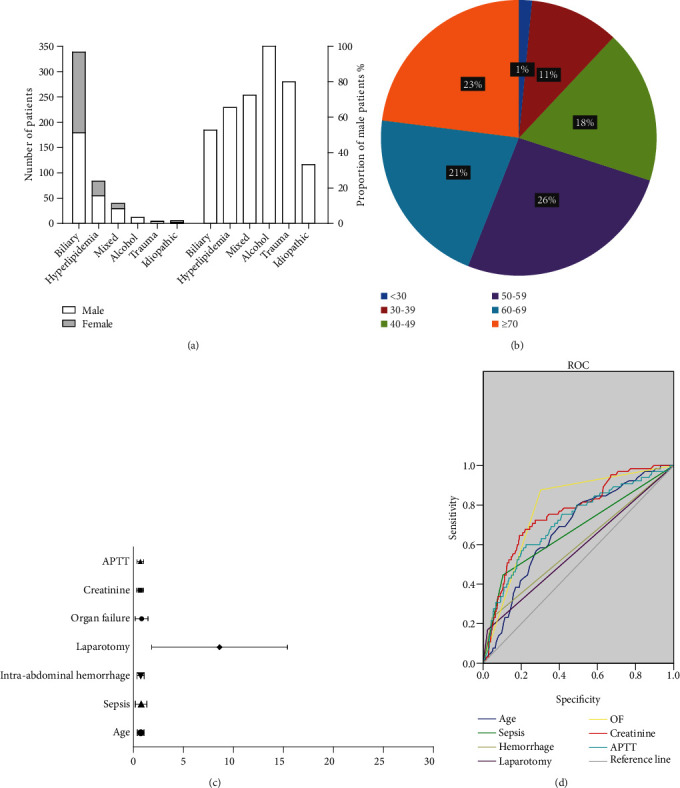
(a) Gender proportion of SAP patients with different etiologies. (b) Age distribution map of all SAP patients. (c) Forest map of risk factors for death in SAP patients. (d) ROC curves for predicting mortality in SAP patients using various variables.

A total of 486 SAP patients were included in the study, of which 420 survived and 66 (13.58%) died. There are 281 male, accounting for 57.82%. The proportion of males with alcoholic pancreatitis or hyperlipidemic pancreatitis is relatively high (Figure 1(a)). There was no significant difference in sex ratio between the survival group and the nonsurvival group (Table 1). The median age of all patients was 49 years old, and the median age of the patients in the nonsurvival group was greater than that in the survival group (56 vs. 46 years old, *P* < 0.01). SAP patients were mainly distributed between the ages of 40-49 (18%), 40-59(26%), 60-69 (21%), and ≥70 years old (23%, Figure 1(b)). The common etiologies of SAP are biliary tract diseases (69.75%) and hyperlipidemia (17.28%). There was no significant difference in the etiology between the nonsurvival group and the survival group. The common comorbidities of SAP patients were hypertension and diabetes, and the morbidity of diabetes in the nonsurvival group was higher than that in the survival group (30.30% vs. 19.05, *P* = 0.04, Table 1). There was no difference in unhealthy lifestyle habits between the two groups of patients.

The most common complications caused by SAP were ARDS (50.62%), AKI (30.45%), sepsis (27.16%), and abdominal fluid collection (27.57%). The incidence of ARDS, AKI, sepsis, septic shock, organ perforation, intra-abdominal hemorrhage, fluid collection, and ACS in the nonsurvival group was higher than that in the survival group (Table 1).

The incidence of organ failure (50.00% vs. 87.88%, *P* < 0.01) and infected pancreatic necrosis (23.10% vs. 43.94%, *P* < 0.01) in the nonsurvival group was higher than that in the survival group (Table 1). Patients in the nonsurvival group required more medical interventions, including mechanical ventilation, vasopressin use, percutaneous puncture drainage, laparotomy, and blood product infusion.

### 3.2. Comparison of Laboratory Examinations between Patients in the Survival Group and the Nonsurvival Group

The leukocyte, neutrophil, and platelet counts in the nonsurvival group were lower than those in the survival group (11.19 ± 6.12 vs. 13.14 ± 6.43, *P* = 0.02; 9.72 ± 5.91 vs. 11.34 ± 5.70, *P* = 0.03; 116.50 (85.00, 190.00) vs. 163.00 (122.00, 217.00), *P* = 0.02). The serum ALT and total bilirubin levels of patients in the nonsurvival group were higher than those in the survival group (40.00 (20.00, 148.00) vs. 23.00 (14.00, 50.00), *P* = 0.04; 24.80 (16.90, 64.65) vs. 19.50 (13.70, 30.10), *P* = 0.01, Table 2). The serum creatinine and LDH of patients in the nonsurvival group were higher than those in the survival group (162.00 (78.50, 259.00) vs. 68.00 (52.00, 99.00), *P* < 0.01; 635.50 (333.50, 1419.75) vs. 374.00 (245.00, 539.00), *P* = 0.03). In terms of coagulation function, APTT was higher in the nonsurvival group than that in the survival group (48.10 (36.90, 69.55) vs. 36.25 (30.20, 44.00), *P* < 0.01), while PTA was lower in the survival group (77.10 (49.40, 96.90) vs. 97.00 (80.88, 111.50), *P* < 0.01). The Ranson score of patients in the nonsurvival group was higher than that in the survival group (4.41 ± 1.46 vs. 3.45 ± 1.34, *P* < 0.01, Table 2).

### 3.3. Binary Logistic Regression Analysis of Differential Indicators between the Two Groups of Patients

We further conducted binary logistic regression analysis on the indicators with differences between the two groups, and the results showed significant differences in age (OR 1.060, 95% CI 1.032-1.088, *P* < 0.01), sepsis (OR 0.147, 95% CI 0.067-0.324, *P* < 0.01), intra-abdominal hemorrhage (OR 0.263, 95% CI 0.096-0.720, *P* = 0.009), laparotomy (OR 6.311, 95% CI 1.793-22.207, *P* = 0.004), organ failure (OR 0.124, 95% CI 0.052-0.297, *P* < 0.01), creatinine (OR 1.003, 95% CI 1.001-1.006, *P* = 0.017), and APTT (OR 0.018, 95% CI 1.004-1.032, *P* = 0.012), as shown in Table 3. Multivariate analysis of other variables showed *P* > 0.05. The forest map of the relationship between various variables and 90-day mortality was shown in Figure 1(c).

### 3.4. The Receiver Operating Characteristic Curve Comparisons of Different Parameters in Predicting 90-Day Mortality

We further evaluated the role of these parameters in predicting the 90-day mortality of SAP patients. These variables in this study were used to predict the mortality of SAP patients, and the AUC were all less than 0.8 (Figure 1(d) and Table 4).

### 3.5. Analysis of Death Causes of SAP Patients

**Figure 2:**
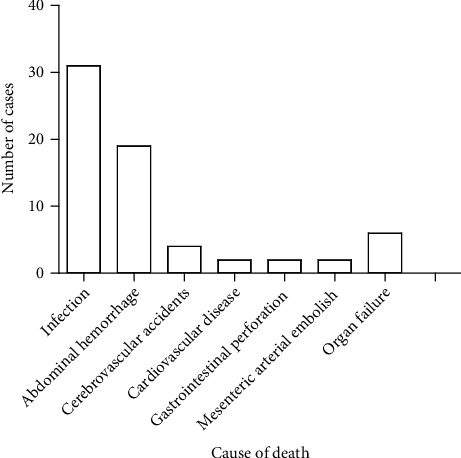
Causes of death in SAP patients.

A total of 66 patients died in this study. The specific causes of death were as follows: infection in 31 cases (46.97%), abdominal bleeding in 19 cases (28.79%), cerebrovascular accident in 4 cases (6.06%), heart disease in 2 cases (3.03%), gastrointestinal perforation in 2 cases (3.03%), mesenteric artery embolism in 2 cases (3.03%), and organ failure in 6 cases (9.09%) (Figure 2).

## 4. Discussion

The study investigated the etiology, severity, and mortality of 486 patients with SAP in Western China. Previous reports have shown that gallstones and alcohol are the most common causes of AP in China [11–13]. In the study, gallstones remained the primary cause of AP, which is consistent with the previous reports [10, 14]. However, the second leading cause of AP in our study was not alcohol, but hyperlipidemia. This result is inconsistent with the reports from other regions in China [11, 13]. We speculate that this difference may be related to the dietary habits of Guizhou Province. Guizhou Province is located in Western China, with a humid and rainy climate. Local people enjoy high-fat and spicy diets. A study in 2017 also showed that the incidence of hyperlipidemic pancreatitis in China has increased year by year [10]. This phenomenon requires the vigilance of clinical doctors and strengthening health education. In the study, there was no significant difference in the etiology between the survival group and the nonsurvival group. Previous studies also have not found a correlation between the etiology and outcome of AP [15, 16].

In the study, SAP patients were mainly distributed in the population over 40 years old. This result may be related to the fact that all patients included in this study were SAP patients. Furthermore, previous studies showed that the incidence rate of biliary pancreatitis usually increased with age [17–19]. In addition, elderly patients may be more likely to progress to SAP [10]. Our study also showed that patients in the nonsurvival group were older than those in the survival group, and multivariate analysis suggested that age is a risk factor for mortality. When suffered from the same level of stress, elderly people are more prone to organ failure and difficult to recover. There was no significant difference in the sex ratio between the nonsurvival group and the survival group. Therefore, gender may not be a risk factor for the severity of SAP patients' condition and mortality.

The 90-day mortality of SAP patients in this study was 13.58%, similar to a previous study (11.1%) [5]. The incidence of complications in patients in the nonsurvival group was significantly higher than that in the survival group, such as ARDS, AKI, sepsis, abdominal hemorrhage, fluid collection, and ACS. The mortality of AP patients with MODS increases more than 100 times [20]. This study also showed that the incidence of organ failure in the nonsurvival group was higher than that in the survival group. Further analysis showed that organ failure was an independent risk factor for death in SAP patients, which was consistent with the previous reports [21]. In addition, necrosis and infectious necrosis are significantly correlated with an increase in AP mortality rate [4, 21]. This study showed that the incidence of infectious pancreatic necrosis in the nonsurvival group was higher than that in the survival group. However, the multivariate analysis did not show that it was an independent risk factor for death in SAP patients, which is different from a previous report [21]. This difference may be related to the fact that all the patients we included were SAP and the number of dead patients was less.

Due to the higher incidence of complications in the nonsurvival group compared to the survival group, patients in the nonsurvival group received more medical interventions, such as mechanical ventilation, vasopressin, percutaneous drainage, laparotomy, and blood transfer products. Regression analysis showed that laparotomy was associated with death. The reason for this result is that SAP patients who need open surgery are more critically ill. There has been substantial evolution of strategies for interventions in recent years, from open surgery to minimally invasive surgical and endoscopic step-up approaches [22]. In clinical practice, there are still some patients who have to undergo open surgery because of their condition.

This study showed that the white blood cell count, neutrophil count, and platelet count of patients in the nonsurvival group were lower than those in the survival group, while ALT, total bilirubin, creatinine, and LDH were higher than those in the survival group. In terms of coagulation function, APTT and TT were higher in the nonsurvival group than those in the survival group, while PTA was lower in the survival group. A multivariate retrospective analysis showed that only APTT and creatinine showed differences between the two groups. A recent retrospective study showed that in multivariate regression, APTT was also found to be a risk factor for death in AP patients [23]. In dogs with AP, serum creatinine > 212 *μ*mol/L was associated significantly with poor prognosis [24]. Multivariate analysis of a previous study has determined that creatinine, glucose, and pleural fluid at admission are independent risk factors associated with postdischarge mortality in AP patients [25]. A multivariate logistic regression indicated that blood urea nitrogen and serum creatinine at 24 hours after hospitalization were independently associated with SAP [26]. Therefore, these results suggest that we should pay attention to the levels of APTT and serum creatinine at the time of admission in SAP patients. When these differential variables were used to predict the mortality of SAP patients, the AUC were all less than 0.8. This result indicates that a single variable does not perform well in predicting mortality in SAP patients. Imaging combined with other variables may perform better in predicting outcomes of SAP patients.

The three main causes of death in SAP patients in this study were infection, abdominal bleeding, and organ failure. Similarly, a previous study showed that the main causes of death in AP patients were organ failure and infectious diseases [27]. A recent study suggests that respiratory failure is the main cause of AP patient death [7]. Previous studies have shown that splenic artery, portal vein, spleen, and peripancreatic vessels are the most common sources of bleeding in AP, with associated mortality rates of 33.3%, 50.0%, 30%, and 28.5%, respectively [28]. Organ failure and infection can also increase the risk of bleeding. Therefore, the main cause of death in SAP patients is pancreatitis-related organ failure and secondary infection.

The limitations of this study are as follows: firstly, it is a single-center study, and the sample size of nonsurvival patients is small, which may lead to no statistical difference in some independent risk factors of death, such as infectious pancreatitis necrosis. Secondly, the laboratory examination results of this study were obtained at the time of patient admission and did not undergo continuous observation. Perhaps, the trend of changes in these variables is better than a single time point in predicting patient prognosis. Thirdly, as this study was a retrospective analysis, some infection indicators were not routinely checked, so important inflammatory indicators such as IL-6 and procalcitonin were missed. Finally, the patients included in this study are SAP patients admitted to ICU, which may lead to selection bias. In addition, our hospital is a referral center, and the SAP patients included are critically ill, which may also lead to selection bias. This selection bias may lead to the results of this study not applicable to all AP patients.

In conclusion, age, sepsis, abdominal hemorrhage, and organ failure are risk factors for death in SAP patients. Patients with SAP who underwent open surgery had a higher mortality rate. SAP patients with high creatinine and prolonged APTT upon admission require doctors to be vigilant. Age, sepsis, organ failure, abdominal hemorrhage, laparotomy, creatinine, and APTT did not perform well in predicting mortality in SAP patients.

## Figures and Tables

**Table 1:** Comparison of clinical characteristics between the survival group and the nonsurvival group in patients with SAP.

	All patients (*n* = 486), *n* (%)	Survivor (*n* = 420), *n* (%)	Nonsurvivor (*n* = 66), *n* (%)	*P*
Gender				0.62
Females	205 (42.18)	179 (42.62)	26 (39.39)	
Males	281 (57.82)	241 (57.38)	40 (60.61)	
Age (years)				<0.01
Median	49	46	56	
Range	7-89	7-89	27-84	
Etiology				
Biliary	339 (69.75)	289 (68.81)	50 (75.76)	0.25
Hyperlipidemia	84 (17.28)	77 (18.33)	7 (10.61)	0.12
Mixed	40 (8.23)	33 (7.86)	7 (10.61)	0.45
Alcohol	12 (2.47)	11 (2.62)	1 (1.52)	0.59
Trauma	5 (1.03)	5 (1.19)	0(0)	0.37
Idiopathic	6 (1.23)	5 (1.19)	1 (1.52)	0.82
Comorbidity				
Hypertension	137 (28.19)	112 (26.67)	25 (37.88)	0.06
Diabetes	100 (20.58)	80 (19.05)	20 (30.30)	0.04
Chronic respiratory diseases	7 (1.44)	5 (1.19)	2 (3.03)	0.24
Chronic renal failure	7 (1.44)	4 (0.95)	3 (4.54)	0.09
Unhealthy lifestyle				
Smoking	221 (45.47)	188 (44.76)	33 (50.00)	0.43
Excessive drinking	65 (13.37)	55 (13.10)	10 (15.15)	0.65
Complication				
ARDS	246 (50.62)	205 (48.81)	41 (62.12)	0.04
AKI	148 (30.45)	102 (24.29)	46 (69.70)	<0.01
Sepsis	132 (27.16)	102 (24.29)	30 (45.45)	<0.01
Organic perforation	13 (2.67)	8 (1.90)	5 (7.58)	0.03
Intra-abdominal hemorrhage	35 (7.20)	20 (4.76)	15 (22.73)	<0.01
Fluid collection	134 (27.57)	108 (25.71)	26 (37.88)	0.02
Paralytic intestinal obstruction	4 (0.82)	3 (0.71)	1 (1.52)	1.00
ACS	28 (5.77)	17 (4.05)	11 (16.67)	<0.01
Organ failure	268 (55.14)	210 (50.00)	58 (87.88)	<0.01
Infected pancreatic necrosis	126 (25.93)	97 (23.10)	29 (43.94)	<0.01
Medical intervention				
Mechanical ventilation	174 (35.80)	121 (28.81)	53 (80.30)	<0.01
CRRT	336 (69.14)	291 (69.29)	45 (68.18)	0.86
Vasopressin	162 (33.33)	110 (26.19)	52 (78.79)	<0.01
Endoscopic therapy	42 (8.64)	34 (8.10)	8 (12.12)	0.28
Percutaneous drainage	180 (37.04)	144 (34.29)	36 (54.55)	<0.01
Laparotomy	21 (4.32)	10 (2.38)	11 (16.67)	<0.01
Blood transfusion products	339 (69.75)	284 (67.62)	55 (83.33)	0.01
Time from onset of symptoms to ICU admission	3.0 (1.0, 4.0)	3.0 (1.0, 4.0)	3.0 (2.0, 5.0)	0.14
90-day mortality	66 (13.58)			

SAP: severe acute pancreatitis; ARDS: acute respiratory distress syndrome; AKI: acute kidney injury; ACS: abdominal compartment syndrome; CRRT: continuous renal replacement therapy.

**Table 2:** Comparison of laboratory examinations at admission between the survival group and the nonsurvival group in patients with SAP.

Indicators (normal range)	All patients (*n* = 486)	Survivor (*n* = 420)	Nonsurvivor (*n* = 66)	*P*
Leukocyte count (3.5 − 9.5 × 10^9^/L)	12.88 (6.42)	13.14 (6.43)	11.19 (6.12)	0.02
Neutrophil count (1.4 − 6.3 × 10^9^/L)	11.12 (5.75)	11.34 (5.70)	9.72 (5.91)	0.03
Lymphocyte count (1.1 − 3.2 × 10^9^/L)	0.92 (0.49)	0.93 (0.49)	0.87 (0.54)	0.39
Platelet count (100 − 300 × 10^9^/L)	158.00 (113.50, 211.00)	163.00 (122.00, 217.00)	116.50 (85.00, 190.00)	0.02
ALT (9-50 U/L)	25.00 (15.00, 54.75)	23.00 (14.00, 50.00)	40.00 (20.00, 148.00)	0.04
AST (15-40 U/L)	42.50 (26.00, 83.75)	40.00 (25.00,70.00)	93.00 (37.50, 283.50)	0.11
Total bilirubin (5-21 *μ*mol/L)	20.00 (13.80, 31.25)	19.50 (13.70, 30.10)	24.80 (16.90, 64.65)	0.01
Creatinine (41-109 *μ*mol/L)	71.00 (54.00, 118.00)	68.00 (52.00, 99.00)	162.00 (78.50, 259.00)	<0.01
Urea nitrogen (2.8-7.2 mmol/L)	5.79 (3.67, 9.54)	5.33 (3.44, 8.61)	10.52 (7.18, 16.90)	0.48
LDH (140-271 U/L)	394.00 (254.50, 587.00)	374.00 (245.00, 539.00)	635.50 (333.50, 1419.75)	0.03
APTT (23.3-32.5 s)	37.20 (30.80, 46.20)	36.25 (30.20, 44.00)	48.10 (36.90, 69.55)	<0.01
TT (14-26 s)	17.10 (15.60, 19.70)	16.90 (15.50, 19.20)	18.80 (16.05, 28.10)	0.06
PTA (70-150%)	95.60 (77.90, 110.00)	97.00 (80.88, 111.50)	77.10 (49.40, 96.90)	<0.01
hs-CRP (0.068-8.200 mg/L)	129.56 (59.81)	130.02 (59.95)	126.65 (59.23)	0.68

SAP: severe acute pancreatitis; ALT: alanine aminotransferase; AST: aspartate transaminase; LDH: lactic dehydrogenase; APTT: activated partial thromboplastin time; TT: thrombin time; PTA: prothrombin time activity; CRP: C-reactive protein.

**Table 3:** Multivariable binary logistic regression analyses for factors independently associated with 90 d mortality in patients with SAP.

Variable	*β*	SE	Wald *χ*^2^	OR	95% CI	*P*
Age	0.058	0.013	18.712	1.060	1.032-1.088	<0.001
Sepsis	1.916	0.402	22.706	0.147	0.067-0.324	<0.001
Abdominal hemorrhage	1.337	0.514	6.759	0.263	0.096-0.720	0.009
Laparotomy	1.842	0.642	8.237	6.311	1.793-22.207	0.004
Organ failure	2.088	0.446	21.916	0.124	0.052-0.297	<0.001
Creatinine	0.003	0.001	5.677	1.003	1.001-1.006	0.017
APTT	0.018	0.007	6.325	1.018	1.004-1.032	0.012

APTT: activated partial thromboplastin time.

**Table 4:** The AUC predictive value of variables in predicting 90 d mortality in patients with SAP.

Variable	AUC	95% CI	*P*
Age	0.678	0.612-0.743	<0.001
Sepsis	0.671	0.591-0.750	<0.001
Intra-abdominal hemorrhage	0.592	0.510-0.673	0.017
Laparotomy	0.573	0.492-0.654	0.059
Organ failure	0.787	0.733-0.842	<0.001
Creatinine	0.761	0.700-0.823	<0.001
APTT	0.717	0.647-0.787	<0.001

SAP: severe acute pancreatitis; APTT: activated partial thromboplastin time.

## Data Availability

All data generated or analysed during this study are included in this published article.

## Abbreviations

SAP: Severe acute pancreatitis
AP: Acute pancreatitis
ARDS: Acute respiratory distress syndrome
AKI: Acute kidney injury
ACS: Abdominal compartment syndrome
CRRT: Continuous renal replacement therapy
ALT: Alanine aminotransferase
AST: Aspartate transaminase
LDH: Lactic dehydrogenase
APTT: Activated partial thromboplastin time
TT: Thrombin time
PTA: Prothrombin activity prothrombin time activity
CRP: C-reactive protein
ROC: Receiver operating curve
AUC: Area under curve.
